# Laser Calorimetry Spectroscopy for ppm-level Dissolved Gas Detection and Analysis

**DOI:** 10.1038/srep42917

**Published:** 2017-02-20

**Authors:** Nagapriya K. S., Shashank Sinha, Prashanth R., Samhitha Poonacha, Gunaranjan Chaudhry, Anandaroop Bhattacharya, Niloy Choudhury, Saroj Mahalik, Sandip Maity

**Affiliations:** 1 GE Global Research, GE India Technology Centre Pvt. Ltd., Bangalore, 560066, India

## Abstract

In this paper we report a newly developed technique – laser calorimetry spectroscopy (LCS), which is a combination of laser absorption spectroscopy and calorimetry - for the detection of gases dissolved in liquids. The technique involves determination of concentration of a dissolved gas by irradiating the liquid with light of a wavelength where the gas absorbs, and measuring the temperature change caused by the absorbance. Conventionally, detection of dissolved gases with sufficient sensitivity and specificity was done by first extracting the gases from the liquid and then analyzing the gases using techniques such as gas chromatography. Using LCS, we have been able to detect ppm levels of dissolved gases without extracting them from the liquid. In this paper, we show the detection of dissolved acetylene in transformer oil in the mid infrared (MIR) wavelength (3021 nm) region.

Analysis of dissolved gases in liquids is of prime importance in a wide array of fields like transformer health monitoring^1^,^2^, food and beverage industries (eg measuring amount of ethanol in wine), oil and gas industry (measuring dissolved methane in crude etc.), etc. In many of these fields chemical and electrical analysis cannot be used. Electrical analysis is ruled out due to harsh environmental conditions and due to safety requirements, while in many cases solid-state gas sensing and chemical methods (like electrochemical methods) do not have enough specificity^3^,^4^. In most cases, gas chromatography (GC) - both offline and exsitu - has been used as the best possible solution. Some form of optical analysis is preferred due to its high specificity and its non-invasive nature^5^,^6^. However, optical spectroscopies suffer from need for line of sight, and if insitu, the liquid should be transparent or substantially transparent to the radiation (wavelengths) used. For liquids like water or oil, finding a wavelength where the gas absorbs strongly while the liquids do not absorb is very difficult. Usually the absorbance of the liquid is much stronger than the absorbance of the gas (at the concentrations that need to be detected). Therefore conventionally, the gases are extracted from the liquids and then analyzed, either using an optical method or using GC.

Transformer dissolved gas analysis (DGA) is the measurement and study of composition and concentration of dissolved gases in insulating transformer oil to determine the type and severity of electrical (or thermal) fault occurrence in a transformer. Case studies have shown that DGA and timely intervention can prevent substantial transformer damage. Usually, DGA involves sampling the oil, extracting the gas and analyzing the composition and concentration of the extracted gas. The gas extraction is usually done using extraction techniques such as vacuum extraction or head space extraction. The analysis is typically done using GC^7^,^8^. The key gases measured are acetylene, methane, ethane, ethylene, carbon monoxide, carbon dioxide, hydrogen and moisture. Extraction of gas leads to certain uncertainties and ambiguities in gas concentration determination^9^,^10^,^11^,^12^. An in-oil measurement without gas extraction will help get rid of these uncertainties.

Here a method of detecting and analyzing dissolved gases in liquids without extracting them is presented. The method, though optical (and therefore is non-invasive and has a high specificity), does not require a line-of-sight and can detect dissolved gases in liquids even when the liquid is substantially non-transparent to the incident radiation.

## Theory of LCS

### Principle

Laser calorimetry spectroscopy is a combination of laser-based optical absorption spectroscopy and calorimetry. Laser-based absorption spectroscopies function on the principle that gases preferentially absorb wavelengths that correspond to transitions between electronic, vibrational, or ro-vibrational levels^13^,^14^. Each gas, therefore, has an absorption fingerprint.

When light of a wavelength corresponding to a transition between quantum states of a gas is incident on the gas, the radiation is absorbed. The amount of radiation that is absorbed depends on the line strength of the transition, the concentration of the gas and the length of the path the light traverses in the gas (according to the well-known Beer-Lambert law).


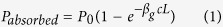


where,

*P*_0_ = Incident laser power

*P*_*absorbed*_ = Power absorbed

*β*_*g*_ = absorption coefficient of dissolved gas

*c* = concentration of dissolved gas in oil

*L* = interaction path length.

Most hydrocarbons, CO and CO_2_, have their fundamental ro-vibrational modes in the MIR region and therefore the detection sensitivity is maximum in the MIR region. The availability of quantum cascade lasers^15^ and MIR detectors has thus enabled very sensitive (sub ppm) detection of these gases.

**Figure 1:**
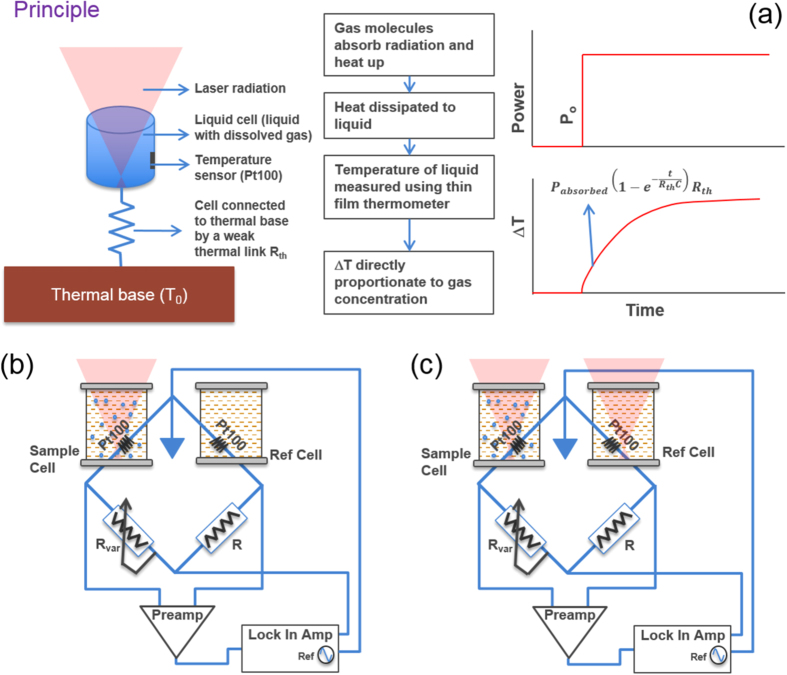
Schematic of the principle of (**a**) LCS (**b**) Differential LCS - used to eliminate the effect of room temperature (**c**) Dual-beam differential LCS that gives the ability to quantify gas concentrations even when the liquid absorbance is significant compared to that of the gas. In differential LCS, the laser beam is incident only on the sample cell, while in dual-beam differential LCS, laser beams are incident on both the sample and reference cells.

In LCS, the rise in temperature due to absorption of radiation is measured. When a sample cell containing the transformer oil with dissolved gases is irradiated with light of a wavelength where a dissolved gas absorbs, the gas absorbs the laser energy and causes heating of the oil (see Fig. 1(a)). If the sample cell of heat capacity *C* is connected thermally to the outside world (or a thermal base) by a link of thermal resistance *R*_*th*_, then the temperature change of the sample due to the gas absorbing the radiation is given by


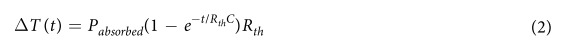


At equilibrium,


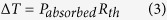


or,


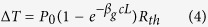


For *β*_*g*_*cL* ≪ 1,


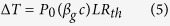


Therefore, the temperature change of the oil is directly proportionate to the concentration of the dissolved gas.

### Eliminating effect of room temperature (Differential LCS)

The temperature that is measured in the experiment is Δ*T* + *T*_0_ (where *T*_0_ is room temperature or the base temperature). The foremost challenge in measurement of gas concentration using LCS is that the temperature change Δ*T* for ppm levels of gas is very small. For example, for acetylene, the highest *β*_*g*_*cL* ≈ 8 × 10^−5^ for 20 ppm concentration (*c*) and 3 mm path length (*L*). (This is about the maximum path length that can be used. The reason for this is explained in the experimental design section). If *P*_0_ = 1 mW and *R*_*th*_ ≈ 5000 K/W, Δ*T* ≈ 0.4 mK. Detection of 0.4 mK when room (base) temperature *T*_0_ is ≈300 K is experimentally challenging and can lead to large errors in the measurement of Δ*T*, thereby leading to erroneous gas concentration estimates. Accurate measurement of Δ*T* (even when the base temperature is 300 K) can be achieved by using a Wheatstone bridge-based differential measurement so that the common mode signal due to room temperature is cancelled out. Figure 1(b) shows a schematic of the differential arrangement used.

Here, *R*_*var*_ is a variable resistor used to balance the bridge such that the output of the bridge is zero when the laser is off. When the laser beam is turned on, the output of the bridge Δ*T*_*measured*_ is the difference between the temperature of the sample (*T*_*sample*_) and the temperature of the reference cell (*T*_*ref*_).


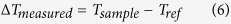



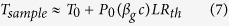



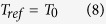


Therefore,


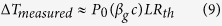


### Dealing with non-transparent liquids (Dual beam differential LCS)

The above equation is valid only when the liquid is transparent to the incident radiation. If the liquid also absorbs,


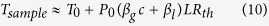


where *β*_*l*_ is the absorption coefficient of the liquid (transformer oil in our case). Therefore,


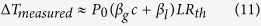


In such a case, gas concentration can be quantified only if *β*_*l*_ is ≪*β*_*g*_*c*, (then the Δ*T*_*measured*_ reduces to that in Equation 9). To be able to quantify gas concentration even when *β*_*l*_ is significant when compared to *β*_*g*_*c*, the dual beam differential method has been developed.

Here, the incident laser beam is split using a beam splitting arrangement such that equal laser powers are incident on both the sample and reference cells **(see** Figs 1(c) and 3(a)). Therefore,


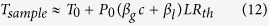



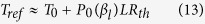


and,


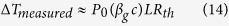


Once again, the measured temperature change is proportionate to the gas concentration.

If the laser powers incident in the two cells are not exactly the same, or if the reference liquid is not the same as the sample liquid, the above equation is no longer valid. Then the experiment is carried out twice - once with the laser wavelength at the absorbance peak of the gas to be detected and the second experiment with the laser wavelength at the gas absorbance minimum. The equations when the incident laser powers are different (*P*_0−*sample*_ is the laser power incident on the sample cell and *P*_0−*ref*_ is the laser power incident on the reference cell) are derived below:

When the laser wavelength is at the gas absorption peak (experiment 1),


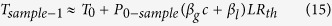



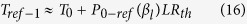



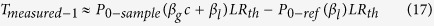


When the laser wavelength is at the gas absorption minimum (experiment 2),


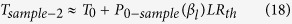



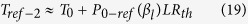



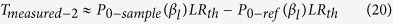


The difference between the two measured temperatures can be used to quantify the gas concentration:


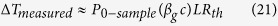


A similar two experiment technique can also be used when the liquids in the sample and reference cell are slightly different. This can happen in the case of transformer dissolved gas analysis as the oil sampled from the transformer can have different absorption properties compared to fresh oil due to aging (since typically fresh oil is used as reference).

Therefore, using the above techniques a gas dissolved in a liquid can be detected even when the liquid is not substantially transparent to the incident radiation, the only constraint being that the liquid does not absorb all of the incident radiation. However, while the basic principle is very straightforward, the experimental implementation is not, and will be discussed in detail in the following sections.

## Experimental Design

LCS is a combination of optical, thermal and electrical detection and involves significant design constraints. The design considerations in the experimental implementation of LCS are explained below. The description below is for the specific case of acetylene in transformer oil. However, the method can easily be extended to other gases dissolved in other liquids.

### Optical

#### Selection of laser lines (wavelength selection)

Transformer oil is chemically very complex and may comprise of as many as 2900 paraffinic, napthenic and aromatic hydrocarbons^16^. It therefore absorbs very strongly across almost the entire range of absorption wavelengths of the dissolved gases to be detected. It is necessary to find a wavelength window where:

- The gas absorbs very strongly while
- The transformer oil absorbance is a minimum.

A proper selection of the line can optimize the accuracy and performance of the sensor.

Line selection for acetylene is straightforward. Several studies have been done on identification of acetylene lines using absorption spectroscopy. Also, the HITRAN database has quantitative spectroscopic parameters for many of the small molecular constituents of atmosphere including acetylene^17^,^18^,^19^,^20^. We have used the database to determine regions where acetylene absorbs very strongly. The fundamental mode for acetylene is between 2950 nm (≈3390 cm^−1^) and 3150 nm (≈3175 cm^−1^) in the MIR region. The largest absorbance peaks occur in the region around 3021.6 nm (3309.5 cm^−1^). This is the best region to detect trace quantities of acetylene.

The absorption spectrum of transformer oil cannot be directly obtained from any database. Several studies have been done to measure the transmission of mineral oil (transformer oil)^21^,^22^,^23^. However, different transformer oils can have different optical properties. Aging can also influence the absorption spectrum of transformer oil. Therefore, the spectrum for the oil being used was experimentally measured using Fourier transform infrared spectroscopy (FTIR).

**Figure 2:**
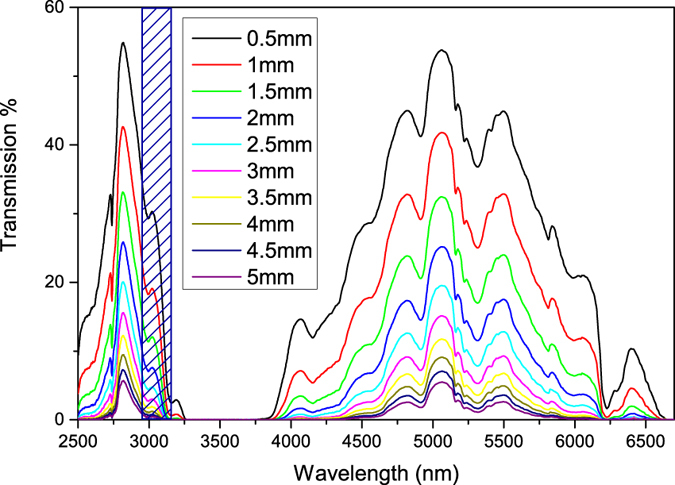
FTIR spectrum of transformer oil in the MIR region for different path-lengths. The blue shaded band is the region of the fundamental vibrational band of acetylene.

Figure 2 shows the FTIR spectrum for transformer oil (TrueNorth Oil Type 1 Naphthenic derivative oil) which we have used for our LCS experiments. The FTIR data was taken for different path lengths of interaction of light with the oil. It can be seen that transformer oil has a broad absorption spectrum. The region where acetylene has its fundamental vibrational band is shown by the blue shaded band. The appropriate laser wavelength is now selected such that the ratio of the absorbance of acetylene to that of the transformer oil is maximum. We find that the region around 3021.6 nm - the wavelength where acetylene absorption is the strongest is best suited for LCS. We have used this wavelength region for detection.

#### Path length selection

As can be seen from Fig. 2, transformer oil absorbs strongly in the region where acetylene absorbs. The strong transformer oil absorbance sets the limit on the pathlength for the system. The pathlength has to be as high as possible so that the signal from acetylene can be maximized. However, the pathlength should be small enough such that the transformer oil does not absorb all of the incident radiation. We find that a pathlength of about 3 mm, such that the absorbance due to the oil is about 60–80%, is ideal for detection of dissolved gas. For a pathlength of 3 mm, if *P*_0_ ≈ 1 mW, Δ*T* due to oil is ≈2 K and Δ*T* due to gas (20 ppm acetylene) is ≈0.4 mK. It can clearly be seen from this example that the temperature change due to oil absorbance is about 4 orders of magnitude larger than that due to gas absorbance. Therefore, careful selection of wavelengths alone is not sufficient to detect trace amounts of fault gases in transformer oil. This is the reason so the dual beam differential arrangement discussed earlier was devised.

### Thermal

The calorimeter has to be designed such that very small concentrations of dissolved gas can be detected within as small a time as possible.

The equilibrium temperature rise is directly proportionate to *R*_*th*_ (as can be seen from equation 5). Therefore the larger the *R*_*th*_, the better the sensitivity (i.e., lower gas concentrations can be detected). However, the thermal time constant of the calorimeter *τ* is the product of the thermal resistance and the heat capacity of the cell + sample system (*τ* = *R*_*th*_*C*). For fast detection, the thermal time constant has to be small. Therefore, to keep sensitivity high and time constant low, the heat capacity of the cell + sample has to be minimized and *R*_*th*_ has to be as high as possible.

To maximize *R*_*th*_, all modes of heat leak - conduction, convection and radiation - to the base have to be minimized.

**Figure 3:**
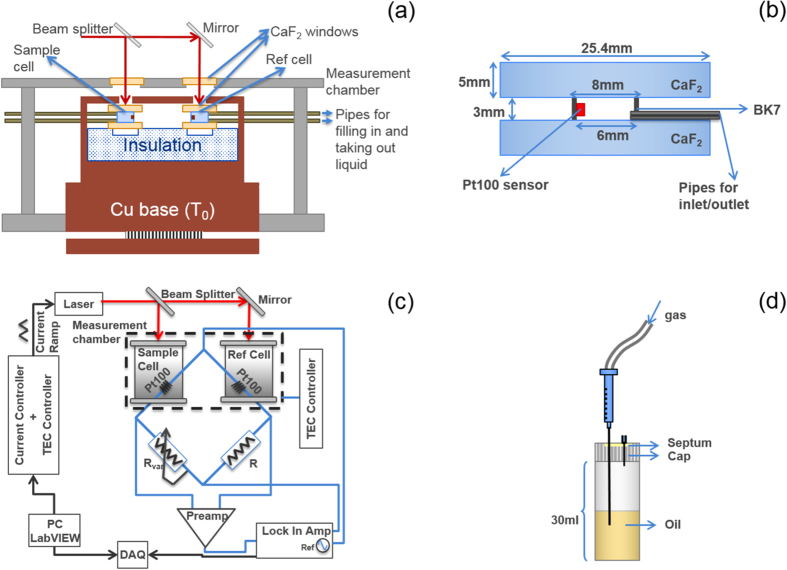
(**a**) Schematic of the experimental arrangement used for LCS (**b**) Sample and reference cell design (**c**) Electronics and the Wheatstone bridge arrangement used to eliminate room temperature fluctuations and other common mode noise and to increase the dynamic range of the measurement (**d**) Bubbling set-up used for the preparation of transformer oil with dissolved gas of a desired concentration.

Conduction occurs through physical contact. The sample and reference cells are therefore isolated from the thermal base using air gaps and low thermal conductivity materials (Fig. 3(a)). Apart from this, the biggest conduction occurs through the electrical leads. The *R*_*th*_ due to conduction by leads can be given by:


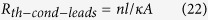


Here *l*: length of the wire; *A*: area of cross-section of the wires; *n*: number of wires and *κ*: thermal conductivity of the material of the wire. To minimize *R*_*th*−*cond*−*leads*_, long, thin phosphor-bronze wires have been used as leads instead of the standard short copper wires. The thermal conductivity of phosphor-bronze is about 8 times smaller than that of copper making the thermal resistance as many times larger. The thermal resistance is further increased by using long and thin wires instead of short thick wires (as *R*_*th*_ is directly proportionate to the length of the wire and inversely to its area of cross-section).

The best way to minimize convection is by keeping the system in vacuum. However, as this would complicate the system design on-field, vacuum is not used.

The other heat leak path is where heat is conducted out to the cell walls and is then lost through convection. As the wall thickness of the cell increases, the heat leak due to conduction decreases while that due to convection increases. Finite element analysis showed that the heat leak from conduction is very small compared to that due to convection. Therefore the smaller the wall thickness, the lower the total heat leak. We have used a wall thickness of about 1 mm.

Radiation heat loss is minimized by making the inside of the copper enclosure shiny. Our simulations show that heat leak due to radiation is significant compared to that due to convection when the temperature rise of the liquid is less than 100 mK. Since the temperature rise for transformer oil is always typically larger than 100 mK, the most significant heat leak occurs through convection from the cell walls.

### Electrical

Measurement of temperature changes of the order of tens of *μ*K requires measurement of voltages of tens of nanovolts. For accurate measurement, a phase sensitive detection using a lock-in amplifier is used here. The details will be discussed in the methods section.

## Methods

### Experimental arrangement

Figure 3(a) illustrates a schematic of the experimental arrangement employed for LCS. The set-up consists of a sample cell and a reference cell mounted on a thermally insulating block. The areas just under the cells have air gaps to increase the thermal resistance further. The insulator sits on a copper thermal base which is in contact with a copper enclosure which surrounds the cells and the insulator. The copper enclosure has a slot through which laser light can enter the sample and reference cells. This whole arrangement is mounted inside a stainless steel chamber for better thermal stability. The temperature of the liquids inside the sample and reference cells are measured using Pt100 thin film thermometers mounted inside the cells. The leads are thermally grounded on the copper base whose temperature is kept constant using a thermoelectric cooler (TEC) controlled by a thermoelectric controller (TED4015, Thorlabs Inc.). Silicone pipes connected to the sample and reference cells are used to fill liquid inside the cells and for draining the liquids out. The different parts of the set-up are explained in detail below.

#### Laser Setup

A room temperature DFB Intraband Cascade Laser (ICL) emitting at 3021 nm (Nanoplus GmbH) is used as the excitation source. The laser is collimated with an aspheric lens (C028TME-E, f = 5.95 mm, NA = 0.56, Mounted Geltech Aspheric Lens, AR: 3–5 *μ*m). A pair of barium fluoride plano-convex lenses (LA0835-E, BaF_2_ Plano-Convex Lens, f = 50.0 mm, AR-Coated: 3–5 *μ*m and LA0737-E, BaF_2_ Plano-Convex Lens, f = 100.0 mm, AR-Coated: 2–5 *μ*m) are used to create a beam size of ≈3 mm. The collimated light beam is aligned such that one part of the beam falls on the sample cell and the other part of the beam falls on the reference cell using a MIR broadband CaF_2_ beam splitter (50:50)(BSW510, Thorlabs Inc) and gold coated mirror (PF10-03-M01, Thorlabs Inc). The laser wavelength can be tuned by changing the laser temperature or the laser current. For LCS, the laser is typically tuned by changing the current with the temperature kept constant. This is achieved using a laser current controller and temperature controller (DCC110, DTC110, Toptica GmbH). The desired wavelength range for the experiment with acetylene in transformer oil is 3021 nm-3022 nm which corresponds to a laser current range of 110 mA–170 mA at a laser temperature of 38 °C. It is typically necessary to pre-calibrate the laser wavelength as a function of the laser current and temperature (this data is also provided by the laser manufacturer) to ensure that atleast one gas transition line (absorption peak) and one off transition line is covered in the wavelength range used.

#### Laser calorimetry cell

The cell design is shown in Fig. 3(b). The cell is made using a cylindrical BK7 glass tube (inner diameter 4 mm and wall thickness 1 mm) covered on both sides using AR coated CaF_2_ windows (WG51050-D, Thorlabs Inc). The cell has inlet and outlet ports for sample injection and removal.

#### Measurement chamber

The measurement enclosure (Fig. 3(a)) is a cylindrical stainless steel chamber (inner diameter ≈23 cm, wall thickness ≈3 mm and height ≈15 cm) with the top plate consisting of a pair of 5 mm thick CaF_2_ windows (WG51050-D, Thorlabs Inc). The top plate is designed such that the CaF_2_ windows align with the sample and reference cells so that collimated laser beams from the beam splitter and mirror arrangement can pass through into the cells. The electrical connections for the TEC and Pt100s are made through BNCs mounted on the circumference of the chamber. The chamber also has a port for the pipes used for injecting and removing the sample to pass through.

#### Temperature measurement and electronics

Platinum thin film temperature sensors (Pt100, 100 Ω at 25 °C, Omega Corporation) are used for temperature measurement. Pt100 sensors are integrated into the sample and reference cells such that they are in good thermal contact with the liquid. The Pt100 sensors form the two upper arms of a Wheatstone bridge arrangement Fig. 3(c).

The bridge arrangement is used to measure the difference in the resistance change between the two Pt100 sensors (in the sample and reference arm) when radiation is incident on the cells. Since the resistance of a Pt100 sensor is a linear function of temperature (with a temperature coefficient of resistance 0.00385/K), the difference in resistance is used to calculate the difference in the temperature change due to gas absorption.

The two lower arms of the bridge have a standard resistor and a variable resistor (precision resistance box HARS-X-5-0.01). The bridge is biased using the ac output (1 kHz, 1 V_*pp*_) of a lock-in-amplifier (SR830, SRS) and balanced using the variable resistor when no radiation is incident on the cells. The output of the bridge is given to a low noise transformer coupled preamplifier (SR554, SRS; 0.1 nV/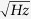 input noise, 0.1 Hz to 40 kHz bandwidth, Gain of 100 or 500). The output of the preamplifier is fed to the lock-in for phase sensitive detection. The first harmonic lock in signal is digitized using a DAQ card and recorded using a personal computer. The voltage variation data is plotted in real time via a LABVIEW (Labview 2011, National Instrument, Austin, TX) software interface. An in-house developed LABVIEW program is used to control the sensor system (laser controller, TEC controller, lock-in amplifier, etc.).

#### Sample preparation

To prepare transformer oil with dissolved gas of a desired concentration, we use an in-house built bubbling set-up as shown in Fig. 3(d). The set-up consists of a gas-tight liquid bottle with a septum. Gas of the desired concentration is bubbled into the oil using a gas-tight syringe. The oil is constantly stirred with a magnetic stirrer to ensure good mixing of the gas. The gas is bubbled at a very slow rate such that the gas comes out of the syringe tip as a bubble. Bubbling gas in too fast can result in the oil coming out of the output port of the bottle). The typical bubbling time is ≈4 h. A portion of the oil is now sampled using a gas-tight syringe and the concentration verified using a photo-acoustic spectroscopy-based off the shelf instrument (TransportX, GE). Once the gas concentration is confirmed, the oil is sampled out and filled into the sample cell for LCS.

### Experimental procedure

The thermal base is set and controlled at a temperature slightly higher than room temperature. The laser is aligned such that the beam falls in the centre of the sample and reference cells. To ensure the best laser alignment, the following procedure is followed: one part of the laser beam is blocked using a beam dump. Now the laser is incident on only one cell. The beam position is adjusted to get maximum signal at the lock-in amplifier. At this position the laser is best aligned with the cell. The same procedure is repeated for the other cell. Now the beam dump is removed and the bridge is balanced with the variable resistor to get the minimum output voltage at the lock-in amplifier.

The sample cell is filled with the sample (oil with dissolved gas of a specific concentration) and the reference cell is filled with the reference liquid. The laser current is set to a value such that the gas does not absorb at the wavelength emitted. The voltage output is measured and recorded continuously as a function of time. This process is repeated for different laser currents (or wavelengths) including wavelengths where the gas absorbs strongly.

**Figure 4:**
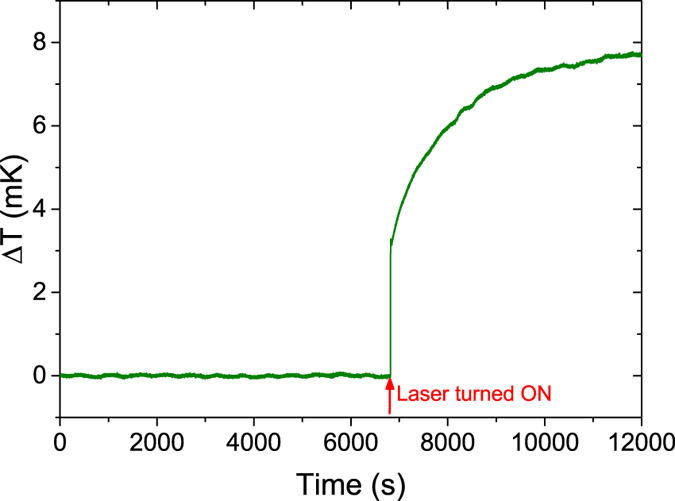
Temperature rise as a function of time for transformer oil with 28 ppm dissolved acetylene. The laser current is 131.6 mA.

The recorded voltage is converted to temperature. Figure 4 shows temperature as a function of time for 131.6 mA current. The exponential rise when the laser is turned on can clearly be seen. The equilibrium temperature rise is measured by fitting equation 2 to the data. The equilibrium temperature rise is measured for the different laser wavelengths to get a plot of temperature rise as a function of wavelength. The measurements are repeated for different concentrations of dissolved gas to get temperature change as a function of concentration. A linear relationship is expected.

## Results

### Baseline noise and Detection limit

**Figure 5:**
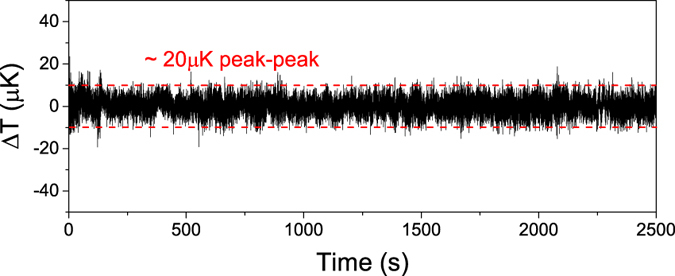
Recorded time-series of baseline temperature fluctuations. Note that the peak-peak temperature fluctuations is ≈20 *μ*K. The fluctuations are white and can therefore be reduced even further by averaging.

The first experiment done was to record, measure and analyze the baseline noise floor which sets the minimum detection limit for LCS. This is done by measuring the baseline fluctuations when there is no liquid in the cells. Figure 5 shows the recorded baseline temperature fluctuations. As can be seen, the fluctuation is ≈20 *μ*K. This corresponds to ≈1 ppm of C_2_H_2_ (for an *R*_*th*_ of 5000 K/W and a laser power of 1 mW).

Lower detection limits can be achieved in two ways: (i) by increasing the thermal resistance which would increase the temperature change (ii) by averaging - the measured thermal fluctuations are white and therefore averaging can decrease this noise, thereby lowering the detection limit.

### Reference measurement - transformer oil with no dissolved gas in both cells

**Figure 6:**
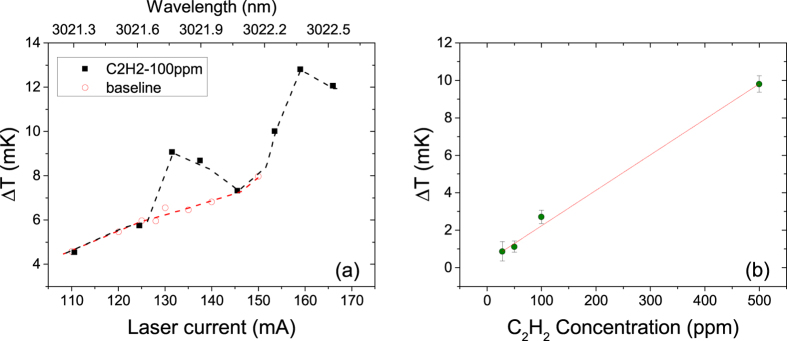
(**a**) Temperature change as a function of wavelength for transformer oil with no acetylene (red, open circles) and transformer oil with 100 ppm dissolved acetylene (black, closed squares). The peak at ≈3021.7 nm is due to dissolved acetylene (**b**) Temperature change at 3021.7 nm as a function of acetylene concentration.

Slight differences in the sample and reference cells and in the incident power on the cells lead to a non-zero Δ*T*_*measured*_ even when the same oil is filled in both the cells. The laser power output changes with change in current. This leads to a non-flat Δ*T*_*measured*_ as a function of wavelength. The Δ*T*_*measured*_ as a function of wavelength for transformer oil in the acetylene detection wavelength range is shown in Fig. 6(a) by open circles (red line). It can be seen that the temperature rise increases with wavelength. This is due to increase in laser power with increasing laser current. This data serves as the baseline for the measurement of dissolved acetylene in transformer oil.

### Dissolved acetylene detection

Figure 6(a) shows a plot of temperature change as a function of wavelength and laser current for 100 ppm of dissolved acetylene in transformer oil (black, closed squares). The plot also shows the reference measurement (in red dashed line). The temperature change due to acetylene for different acetylene concentrations at ≈3021.7 nm wavelength is shown in Fig. 6(b). The linearity in temperature rise with concentration is evident. It can clearly be seen that we have experimentally detected 28 ppm dissolved acetylene in transformer oil. LCS, therefore is suitable for measuring dissolved gases in liquids.

## Discussion

The experimentally achieved minimum detection limit for acetylene in transformer oil is ≈28 ppm which corresponds to a fractional absorbance of ≈10^−4^. With the current system, detection of 10 ppm (fractional absorbance of ≈4 × 10^−5^) is possible. This limit has not been experimentally validated due to constraints in the gas dissolving process. In-gas detection techniques such as tunable diode laser absorption spectroscopy (TDLS) have a minimum detection limit corresponding to an absorbance of ≈5 × 10^−5 ^24,25. The fact that LCS, which is an in-liquid detection technique, can achieve similar detection limits makes LCS a promising technique for in-liquid dissolved gas detection.

The detection limit set by the baseline temperature fluctuation corresponds to an absorbance of ≈4 × 10^−6^ for incident laser power of 1 mW. In the current setup, the laser power incident on each cell is only 0.5 mW and therefore the detectable fractional absorbance limit is 8 × 10^−6^. However, the fractional absorbance corresponding to the achieved detection (28 ppm) is ≈10^−4^ with the possibility of going down to a fractional absorbance of 4 × 10^−5^. This is about five times larger than the limit set by baseline temperature fluctuation. The reason for this discrepancy is the presence of temperature oscillations due to changes in room temperature. We found that the air-conditioning caused oscillations in temperature. The amplitude of the temperature oscillations were ≈0.1 mK making the minimum detectable fractional absorbance 4 × 10^−5^. Better temperature control can lower the limit of detection. MIR ICLs with high powers of 10–20 mW are now commercially available. This can further better the limit of detection enabling sub-ppm detection of acetylene in transformer oil.

A comparison of LCS with commonly used techniques for DGA is shown in Table 1. Other techniques such as FTIR and Raman Spectroscopy have very poor sensitivities and detection limits and are not included in the table. It can be seen that LCS can be a potential technique for detection and analysis of dissolved gases.

In conclusion, the newly developed technique of LCS has been described and its application in the detection of dissolved acetylene in transformer oil has been demonstrated in the manuscript. This technique can be expanded to other dissolved gases in oil and to other fields such as food and beverage industry (for assessing oil quality, determining amount of ethanol in wine, soda quality control), oil and gas industry (determining dissolved gases in crude, Pressure-Volume-Temperature simulation), water industry (determining dissolved gases in drinking water, determining biological oxygen demand), etc. where concentration of dissolved gases in liquids need to be measured.

Though there is a long way to go, it is clear that this technique has several advantages over existing methods. First, this is a direct in-liquid, *in-situ* measurement and does not require any gas extraction, thereby eliminating the errors associated with calculating the gas concentration using partition coefficients^9^,^10^,^11^,^12^. The only input to the gas concentration calculation algorithm is pre-calibration with known quantities of dissolved gas. Second, LCS has the ability to measure gas concentrations with a fractional absorbance of 8 × 10^−6^ for an input power of 1 mW and also has an extremely high dynamic range of 10^5^. Third, this technique also enables detection of dissolved gas concentration in highly absorbing liquids due to the dual beam differential measurement.

The key limitations of this technique are the long measurement times (few hours per measurement) and the highly stable environmental temperature control required if very low detection limits are to be reached. This makes the technique cumbersome for use in industrial environments. However, fast measurement methods are being explored currently to make the technique more robust. It must also be mentioned here that since transformer oil is chemically very complex, unknown degradation products might lead to some interference if they have absorbances in the range of the gases being studied. It would be critical to investigate different transformer oils and thier degradation products to select an appropriate wavelength for analysis. At present LCS is a very sensitive, in-liquid lab measurement technique for dissolved gas detection.

## Additional Information

**How to cite this article:** K. S., N. *et al*. Laser Calorimetry Spectroscopy for ppm-level Dissolved Gas Detection and Analysis. *Sci. Rep.* **7**, 42917; doi: 10.1038/srep42917 (2017).

**Publisher's note:** Springer Nature remains neutral with regard to jurisdictional claims in published maps and institutional affiliations.

## Figures and Tables

**Table 1:** Comparison of commonly used optical and chromatographic techniques for DGA with LCS.

	Sensitivity^*^	Detection limit^*^	Selectivity	Accuracy	Repeatability	In-oil detection
Gas chromatography^#^	0.1 ppm	0.5 ppm	Good	0.5 ppm	Good	No
PAS (Broadband)^#^	0.1 ppm	0.5 ppm	Fair	0.5 ppm	Good	No
PAS (laser-based)^**^	0.1 ppm	0.5 ppm	Good	0.5 ppm	Good	No
NDIR^#^	0.5 ppm	0.5 ppm	Fair	0.5 ppm	Good	No
LCS	1 ppm^+^	10 ppm^+^	Good	1 ppm^+^	Good	Yes

PAS - Photoacoustic Spectroscopy.

^*^For acetylene; ^#^Commercially available; ^**^Measured in lab.

^+^Detection of 28 ppm has been demonstrated. With the current system, detection of 10 ppm is possible. The baseline noise at the current laser power (1 mW) corresponds to a detection limit of about 1 ppm.
